# Does cultural practice affects neonatal survival- a case control study among low birth weight babies in Aceh Province, Indonesia

**DOI:** 10.1186/1471-2393-14-342

**Published:** 2014-09-30

**Authors:** Rosnah Sutan, Satrinawati Berkat

**Affiliations:** Community Health Department, Universiti kebangsaan Malaysia Medical Centre, Jalan Yaakob Latif, Bandar Tun Razak Cheras, 56000 Kuala Lumpur, Malaysia; Maternal and Child Health Program, Aceh Besar District Health Office, Aceh Province Kragujevac, Indonesia

**Keywords:** Maternal factors, Low birth weight, Neonatal mortality

## Abstract

**Background:**

Cultural practice have often overlooked when providing maternal and child health care services. Low birth weight is the second cause of neonatal mortality in the world but it is a major factor in a developing country such as Indonesia. The purpose of this study is to predict the neonatal mortality among low birth weight babies in Aceh Province Indonesia.

**Methods:**

Unmatched case control study was conducted using data from year 2010 to 2012 in 8 selected districts of Aceh Province Indonesia. A total of 500 samples were obtained. There were 250 of the samples died in neonatal period (case group) and 250 who were alive (control group). There were 26 variables studied and were grouped into 4 factors: neonatal factor, maternal factor, maternal and child health services and neonatal care practices. The data was analysed using bivariate logistic regression and multivariate logistic regression.

**Results:**

There were 13 out of 26 variables found as determinant factors of neonatal mortality among low birth weight babies in Aceh Province. The predictors found in this study were: boy (aOR1.80, 95% CI: 1.09-2.96), moderate low birth weight (aOR17.84, 95% CI: 6.20-51.35), preterm (aOR1.84, 95% CI: 1.07- 3.17), presence of maternal illnesses (aOR1.87, 95% CI: 1.06-3.30), too short or too long birth interval (aOR1.80, 95% CI: 1.20-2.91), inappropriate antenatal care (aOR2.29, 95% CI: 1.34-3.91), inappropriate neonatal visit (aOR7.04, 95% CI: 3.67-13.49), not practicing kangaroo mother care (aOR15.32, 95% CI: 2.85-82.56), not using warm bottle padding (aOR20.70, 95% CI: 6.32-67.80), not practicing ‘*didaring*’ (aOR4.33, 95% CI: 1.83-10.19), late initiation of breastfeeding (aOR2.03, 95% CI: 1.09-3.80), discard colostrums (aOR3.53, 95% CI: 1.93-6.43) and not practicing exclusive breastfeeding (aOR5.58, 95% CI: 2.89-10.77).

**Conclusions:**

Cultural practices are strongly seen among Acehnese. Inappropriate antenatal care and neonatal care, late initiation of breastfeeding, discarding colostrums and not practicing exclusive breastfeeding were related to cultural practices. Improving knowledge heat preservation to prevent hypothermia using Kangaroo mother care, warm bottle padding and ‘*didaring*’ were proven methods to reduce neonatal mortality. Strengthening of health services in screening for high risk cases and anticipate intervention tailored to cultural practices are important to decrease neonatal mortality among low birth weight.

**Electronic supplementary material:**

The online version of this article (doi:10.1186/1471-2393-14-342) contains supplementary material, which is available to authorized users.

## Background

Cultural practice is one of the important factors that the healthcare workers need to focus when providing maternal and child health care services. It involved the management of most illnesses at any stages for an individual who lives in society with strong cultural beliefs. Many studies done earlier had shown the association between cultural practice, its shared beliefs and norms that influence family behaviours in obtaining maternal and child health care [1–3]. Modern healthcare practice is not well accepted or utilized if family awareness and knowledge level is inadequate for them to make good decision. Even though the young are not keen to practice cultural practice, they have no choice but to follow since they live with their family and especially when the bonding as part of extended family is strong. They do that for the purpose of not offending their parents or society. Scarce researches are available focusing on neonatal health and cultural practice. Many cultural practices are still commonly practice without knowing its existing health benefits or potential harm. The common reasons for cultural practicing were due to self-belief, convenience, family pressure and to please the elders [1, 4].

Developing countries are rich with cultural practice for healing of diseases and care of mothers and their newborns [4–9]. A mother who has just delivered her baby is considered as entering into ‘cold’ period and she is vulnerable to infections and exposed to diseases. The mother strongly needs to have adequate rest after delivery and early clinic appointment given is commonly not routinely followed. If the healthcare worker visits her at her house, the family will appreciate. However, they will not bring the mother or baby out of the house during the ‘cold’ period. The healthcare worker needs to inform and educate them the dangerous situation when the baby is especially born with low birth weight. This depended on the capability of healthcare services to provide enough manpower to do routine postnatal visit.

Many babies were reported dead when born with low birth weight [10, 11]. Early neonatal deaths account for 75% of all neonatal deaths, and preventing these depends on the attention to the causes of death that are unique to the first week of life, particularly birth asphyxia and prematurity [12]. World health organization estimates 18 million babies will be born with low birth weights every year with majority in South Asia [12–14]. The fourth Millennium Development Goal (MDG4) has put a target to reduce less than 5 year mortality and its main effort is to focus on preventing neonatal deaths [10–14]. Low birth weight babies survived poorly compared to normal weight babies. Low birth weight (LBW) is defined as a baby born with a birth weight of less than 2500 grams. Even though LBW is not the immediate causes of mortality, it is a major contributor and may jeopardize the newborn’s chance of survival [12–19]. The prevalence rate of LBW worldwide between 2007 and 2011 was 15% of all live births [10]. The highest neonatal mortality among the South East Asian countries is Indonesia [10]. The percentage of LBW in Indonesia in 2010 was 11.1% of live births [20]. A large number of LBW babies contribute to higher neonatal mortality rate (NMR). Among the causes of neonatal mortality around the world between 2007 and 2011 were: preterm birth complications (35%), complications during birth (23%), infection (sepsis, meningitis, tetanus) (15%), pneumonia (11%), congenital abnormalities (9%), diarrhea (2%) and other condition (6%) [10]. NMR in Indonesia in year 2011 was 15/ 1000 live births [20]. The LBW was the second main causes of early neonatal mortality in Indonesia, after respiratory disorders [21–23]. In Indonesia, Aceh Province has reported high prevalence of neonatal deaths especially among the low birth weight babies [20–23]. Aceh province has recorded 40 per 1000 live births which are above the Indonesia national average [24].

LBW babies are vulnerable to risk and there are many health problems related to LBW as compared to baby born with normal weight. Effort in reduction of maternal mortality and morbidity has shown improvement of child survival. Extra effort to improve feeding, attention to warmth and early treatment of infection was noted effective in neonatal death reduction [25–30]. Babies whose mothers died during the first six weeks of their lives have lesser chance of survival [25]. Health disparity is one of important factors that determine neonatal survival [26, 27]. A three delay model described by Thaddeus and Maine [31] for maternal deaths can be applied to prevent neonatal death. It covers delay in recognition of illness, delay in seeking and accessing care, and delay in the provision of care once at a health facility. A study has reported that more than 90% of post-neonatal children with pneumonia were taken for outside care, whereas only 60% of newborn babies with severe infection were taken out of the home for care [30]. Neonatal period is a vulnerable time and should merit the highest-priority attention when responsible governments are making decisions about laws, policies, programs and money [32, 33].

In order to achieve the MDG4 target in reduction under 5 year child mortality, efforts are planned to improve care at neonatal period. Attempts to reduce the proportion of babies born with low birth weights at the population level, in general, have been met with little success [32–35]. However, most deaths in moderately preterm babies and in those born at term but whose growth have been restricted in utero can be prevented with extra attention to warmth, feeding, and prevention or early treatment of infections [26, 27]. Therefore, community practices in neonatal care must be explored further before initiating any intervention plan. Information related to healthcare acceptance and barrier during neonatal period is not routinely collected. There are many factors associated with mortality among the LBW and it can be grouped into maternal factors, nutritional factors, social factors, environmental factors, medical factors, lifestyle, cultural practice and health care factor. Therefore the purpose of this study is to predict the neonatal mortality among low birth weight babies in Aceh Province Indonesia by focusing on its cultural practices and other factors such as maternal, neonatal, healthcare services and homecare practice.

## Methods

An unmatched case control retrospective study was conducted in Aceh Province. Aceh Province is one of 33 provinces in Indonesia. Aceh province is approximately 58,375.63 Km^2^, consists of 23 regencies/districts, 276 sub district and 6455 villages. Population density in Aceh Province is 74 inhabitants/Km^2^[23]. The total population of Aceh province, is about 4.5 millions with the rate of population growth is 1.46% per years. Aceh province has same common health problems with other provinces in Indonesia with high maternal mortality rate, high infant mortality rate, malnutrition problems in infants and pregnant women and chronic and acute infection diseases [20]. Aceh is a multicultural province. Variety ethnic groups with cultural diversity created non homogenous complex cultural of Acehnese. With regard to inheritance practices that women inherit the house while men inherit the land [36]. Ideally, the houses of women and their daughters are located next to each other like matrilineal clusters.

The number of neonatal deaths in Aceh Province in 2010 was 735 of 99,924 live births (0.73% of total live births), neonatal mortality rate 7.3 / 1,000 live birth and the number of neonatal death due to LBW is 217 or 29.5% of all neonatal death [21]. There are 292 community healthcare centres (*Puskesmas)* serving at sub district level. The community midwives (*Bidides*) who are responsible for MCH service in the village was 4,477 out of 6,265 villages (71.4%). There are 26 government hospitals, 21 private hospitals and a thousand small private delivery clinics [20].

Selection of study sites depended on several basic criteria: high level of neonatal mortality, geographical accessibility and security. Eight districts were selected randomly from a list of districts with high level of neonatal mortality (Table 1). High level of neonatal mortality among low birth weight babies was calculated based on Aceh province estimation of 11.1%. Geographical accessibility and security is defined as availability of road for transportation and safe accommodation to be reach by the researchers. The target population of this study was low birth weights babies who were born alive between 2010 and 2012 regardless they survived or died during neonatal period. The inclusion criterion was babies born alive with birth weight between 1,000 and 2,499 grams, in the period of 2010–2012, in study sites and living with biological mother during neonatal period. The exclusion criteria was the presence of congenital abnormalities, multiple birth, delivery by traditional birth attendant and the baby who did not received neonatal visit by health care worker in neonatal period and baby which was hospitalized during neonatal periods (hospitalized more than 14 days in neonatal period) and were adopted baby.

**Table 1:** **Distribution of neonatal deaths among low birth weight in 8 districts**

District	Number of Live Birth (2009–2010)	Estimation of LBW (11.1%)	Number of neonatal deaths due to LBW (2009–2010)	Distance from capital province to capital district (Km)	Road to capital district and sub district
Aceh Besar	16,708	1,854	43	80	Very good
Pidie	18,628	2,067	21	120	Very good
Lhoksumawe	3,189	353	14	280	Very good
Aceh Utara	6,037	670	12	380	Very good
Aceh Timur	8,376	930	35	450	Very good
Aceh Tamiang	3,703	411	32	500	Very good
Bener Meriah	5,127	569	25	300	Under construction
Aceh Tengah	8,376	930	6	320	Under construction
TOTAL	70,142	7,784	188		

Source: Aceh Province Health Office, 2010 & 2011.

The sample size was determined based on the previous study [37], which was related to neonatal mortality among LBW. It was calculated by Power and Sample Size 2 (PS2) based on Fleiss JL formulae [38]. The calculation used the value of alpha =0.05, power =0.08, probability of exposure in the control (P_0_ = 44), probability of exposure in case (P_1_ = 56) and the ratio of controls to cases as 1:1. The total sample size needed was 518 and was divided into two groups for case and control group. The case group was low birth weight babies who died in neonatal period and the control group was the low birth weight babies who survived in neonatal period.

Descriptive and analytical statistics were computed using Statistical Package for Social Sciences version 20.0. Frequency distribution of each variable studied was conducted to check for missing data and normality. Crude odds ratio with 95% confidence interval was performed to assess the association between socio demographic factor, neonatal factor, maternal factor, maternal and child health services factor and neonatal care practices and the neonatal deaths. The association was set as p < 0.05. All explanatory variables that were found associated (p < 0.05) in the bivariate analysis were included in the multivariable logistic analysis. In multivariable logistic regression analysis, variables with p-value <0.05 were identified as predictors of neonatal deaths. The enter method was used in analysis and the −2 log-likelihood ratio test was used to test the overall significance of the predictive equation. Enter method was used referring to the method in SPSS in multiple regression of entering all variables together regardless of significance levels. Confidence interval (CI) and crude odds ratio (OR) was stated. Adjusted odds ratio (aOR) was adjusted with all variables used in this study.

The significance of the variables in the model was assessed by the Wald *χ*^2^ test and CIs. The fit of the model was assessed by the Hosmer-Lemeshow goodness of fit *χ*^2^ test. Variables that were significantly associated with the outcome in bivariable analysis, but not in multivariable analysis were presented together for comparison. Ethical clearance was obtained from UniversitI Kebangsaan Malaysia research and innovation ethic committee. Formal letter of permission was written to Ministry of Health Aceh province, Indonesia before conducting the study. Respondents who gave oral consent were witnesses by the healthcare worker who accompanied the researchers. The witnesses signed the consent form in front of the respondent after explanation given and verbal approval obtained. Most respondents (97%) gave written consent to participate in this study and the rest gave verbal consents as they are not good in writing and reading.

Inappropriate antenatal care in this study means a mother who received antenatal care visit less than 4 times during her antenatal period [39]. Inappropriate referral was defined as a baby delivered at home or in a non-health facility and was not referred to health facilities for identified risk health condition. In appropriate neonatal visit means a neonate born alive but did not obtain minimum standard of scheduled neonatal visit for health examination by the healthcare worker. Inappropriate umbilical cord care is defined as care of umbilical stump using unhygienic ways such as applying traditional ingredients or alcohol. Delay time of first bath is defined as bathe within less than 12 hours after birth. Late initiation of breastfeeding means the baby was breastfed after 1 hour delivery.

**Figure 1:**
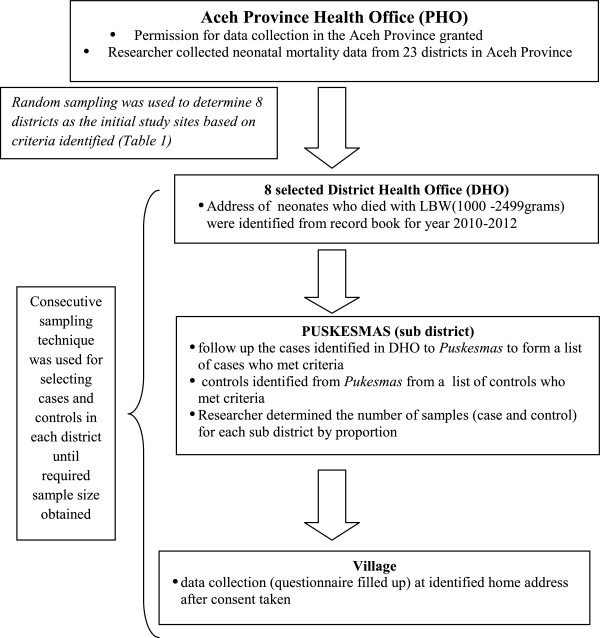
**Flowchart of sampling frame.**

Data collection was carried out from June 2012 – Feb 2013. Cases were universally selected from a list of neonatal deaths recorded in the health districts office chosen (Figure 1). The selected cases health records were tracked down from health clinics. Cases who met the criteria were chosen for follow up at clinic visit to reach the mother or do house visit to complete the questionnaire after consented. Controls were chosen from child health clinic visits. Recall bias on specific information were overcome by checking mother and baby record. Information for practices done during neonatal period were reassess using open ended question to check for consistency in answer given. There were 2 interviewers were trained to ask respondents and completed the questionnaire. Both interviewers were assigned to specific 4 districts each and have to collect for both cases and controls. The questionnaire used was in bilingual English and Indonesia language and were validated during pre-test. The value of cronbach’s alpha obtained was between 0.70 – 0.79. In reporting this study, guidelines from Strengthening the Reporting of Observational Studies in Epidemiology (STROBE) group were followed (Additional file 1).

## Results

There were 250 cases of low birth weight babies died during neonatal period and 250 cases for control group who survived during neonatal period. Both group were comparable for family income (p > 0.05) and distance from home to capital province or district (p > 0.05). There were 4 components on determinant factors used as independent variables: neonatal factor, maternal factor, maternal-child health services and neonatal care practice. Results were tabulated as shown in Tables 2 and 3. Neonatal factor were assessed using sex, birth weight and gestational age at delivery. Majority of cases were baby boys (59.6%) compared to controls (44.0%). There were 31.6% of cases and 2.8% of control were born with weight between 1001 g and 1499 g (moderate low birth weight). There were 82.4% of cases and 58.8% of control were born as pre term with gestational week less than 37 weeks.

**Table 2:** **Results of regression analysis on neonatal and maternal factors of neonatal mortality among low birth weight**

Variables	Case N = 250 (%)	Control N = 250 (%)	Crude OR (95% CI)	p-value	Adjusted OR (95% CI)	p-value
**Neonatal factors**						
**Sex**						
boy	149 (59.6)	110 (44.0)	1.87 (1.31-2.67)	<0.01	1.80 (1.09-2.96)	0.02
girl	101 (40.4)	140 (56.0)	1		1	
**Birth weight (g)**						
1001–1499	79 (31.6)	7 (2.8)	16.00 (7.22-35.5)	<0.01	17.84 (6.20-51.35)	<0.01
1500-2499	171 (68.4)	243 (87.2)	1		1	
**Gestational age**						
Pre term	206 (82.4)	147 (58.8)	3.28 (2.17-4.95)	<0.01	1.84 (1.07-3.17)	0.03
Full term	44 (17.6)	103 (41.2)	1		1	
**Maternal factors**						
**Maternal death**						
Mother dead	0 (0.0)	2 (0.8)	Cannot be estimated			
Mother alive	250 (100.0)	248 (99.2)				
**Maternal illness**						
present	87 (34.8)	39 (15.6)	2.88 (1.87- 4.43)	<0.01	1.87 (1.06-3.30)	0.03
absent	163 (65.2)	211 (84.4)	1		1	
**Maternal age (years)**						
<20 or >35	68 (27.2)	39 (15.6)	2.0 (1.30- 3.14)	<0.01	1.5 (0.92- 2.45)	0.10
20 - 35	182 (72.8)	211 (84.4)	1		1	
**Birth Interval (years)**						
<2 or >4	85 (34.0)	49 (19.6)	2.11 (1.40- 3.17)	<0.01	1.8 (1.20- 2.91)	<0.01
2 - 4	165 (66.0)	201 (80.4)	1		1	
**Parity**						
Para 1 or >5	116 (46.4)	103 (41.2)	1.23 (0.86- 1.76)	0.24	1.1 (0.75- 1.67)	0.56
Para 2-4	134 (53.6)	147 (58.8)	1		1	
**Maternal education level**						
primary	221 (88.4)	214 (85.6)	1.28 (0.75- 2.16)	0.35	1.12 (0.43-3.41)	0.54
≥secondary	29 (13.6)	36 (14.4)	1		1	
**Antenatal care**						
Appropriate	140 (56.0)1	205 (82.0)4	1	<0.01	1	<0.01
Inappropriate	10 (44.0)	5 (18.0)	3.57 (2.38-5.38)		2.29 (1.34-3.91)	
**Place of delivery**						
Non health facilities	105 (42.0)	80 (32.0)	1.53 (1.03-2.21)	0.02	1.03 (0.87-1.98)	0.08
Health facilities	145 (58.0)	170 (68.0)	1		1	
**Birth attendant**						
TBA	22 (8.8)	8 (3.2)	2.91 (1.27-6.68)	0.11	1.23 (0.86-1.76)	0.26
Trained personnel	228 (91.2)	242 (96.8)	1		1	
**Mode of delivery**						
Assisted or LSCS	37 (14.8)	42 (16.8)	0.92 (0.57-1.47)	0.86	0.69 (0.21-2.25)	0.54
Normal delivery	213 (85.2)	208 (83.2)	1		1

**Table 3:** **Results of regression analysis on maternal-child services and neonatal care practice factors of neonatal mortality among low birth weight**

Variables	Case n = 250 (%)	Control n = 250 (%)	Crude OR 95% CI	p-value	Adjusted OR 95% CI	p-value
**Maternal-child health services**						
**Referral**						
Appropriate	155 (62.0)	101 (40.4)	1	0.67	1	0.77
Inappropriate	95 (38.0)	149 (59.6)	0.86 (0.69-1.23)		0.93 (0.02-5.92)	
**Neonatal visit**						
Appropriate	198 (79.2)	239 (95.6)	1	<0.01	1	<0.01
Inappropriate	52 (20.8)	11 (4.4)	10.15 (5.47-18.84)		7.04 (3.67-13.49)	
**Neonatal care practice**						
**Time of first bath**						
Immediately	16 (6.4)	5 (2.0)	3.35 (1.21-9.29)	0.02	2.03 (0.24-16.96)	0.51
Delay (≥12 hours)	234 (93.6)	245 (98.0)	1		1	
**Bath twice a day**						
No	246 (98.4)	237 (94.8)	1	0.04	1	0.65
Yes	4 (1.6)	13 (5.2)	0.30 (0.09-0.92)		1.55 (0.23-10.45)	
**Kangaroo mother care (KMC)**						
Not practice	248 (99.2)	206 (82.4)	26.49 (6.35-110.57)	<0.01	15.32 (2.85-82.56)	0.01
Practice	2 (0.8)	44 (17.6)	1		1	
**Use warm bottle pack**						
Not practice	241 (96.4)	140 (56.0)	21.04 (10.34-2.83)	<0.01	20.70 (6.32-67.80)	<0.01
Practice	9 (3.6)	110 (44.0)	1		1	
**Use lamp bulb**						
Not practice	241 (96.4)	211 (84.4)	3.33 (0.97-11.42)	0.06	0.42 (0.10-1.70)	0.23
Practice	9 (3.6)	39 (15.6)	1		1	
**Use ‘** ***didaring*** **’**						
Not practice	225 (90.0)	147 (58.8)	3.32 (2.00-5.50)	<0.01	4.33 (1.83-10.19)	<0.01
Practice	25 (10.0)	103 (41.2)	1		1	
**Umbilical cord care**						
Appropriate	230 (92.0)	228 (91.2)	1	1.00	1	1.00
Inappropriate	20 (8.0)	22 (8.8)	0.99		0.99	
**Hand wash before touch baby**						
No	247 (98.8)	238 (95.2)	4.51 (1.16-14.89)	0.06	6.62 (0.16-28.41)	0.32
Yes	3 (1.2)	12 (4.8)	1		1	
**Initiation of breastfeeding**						
Late (>1 hour)	219 (87.6)	131 (52.4)	6.42 (4.09-10.07)	<0.01	2.03 (1.09-3.80)	0.03
Early	31 (12.4)	119 (47.6)	1		1	
**Discard colostrums**						
No	119 (47.6)	224 (89.6)	1	<0.01	1	<0.01
Yes	131 (52.4)	26 (10.4)	9.48 (5.89-15.27)		3.53 (1.93-6.43)	
**Exclusive breastfeeding (n = 462)**						
No	195 (92.0)	117 (46.8)	13.0 (7.8-22.0)	<0.01	5.58 (2.89-10.77)	<0.01
Yes	17 (8.0)	133 (53.2)	1		1	

There were 6 variables grouped under maternal factor: maternal death, maternal illness, maternal age, birth interval, parity and maternal education level. None of the cases had maternal death and only 2 controls had maternal deaths (0.8%). There were 34.8% cases with maternal illnesses compared to 15.6% controls. Maternal illness was defined as a mother who was ill by any cause (during antenatal or postnatal) and could not care for her neonate. Young mother (<20 years old) and older mother (>35 years old) group was higher among cases (27.2%) compared to controls (15.6%). Birth interval of less than 2 years or more than 4 years was noted higher among cases (34.0%) compared to controls (19.6%). There is not much different in assessment of parity for both groups (46.4% vs 41.2%). Percentages of low maternal education (primary) were not much differing between the cases and controls (88.4% vs 85.6%). Percentage of inappropriate antenatal care of less than 4 times [39] was higher in case group (44.0%) compared to control group (18.0%). Higher percentages of cases were delivered in non-health facilities (42.0%) compared to controls (32.0%). Non health facilities were referred to either home or at village health post volunteer (*Posyandu*) [40]. Number of babies delivered by traditional birth attendance (TBA) was higher in case group (8.8%) compared to controls (3.2%). Not much difference noted for mode of delivery. There was 85.2% cases had normal delivery compared to 83.2% controls.

Maternal and child health services received by respondents were assessed based on 2 items: referral and neonatal visit. Referral in this study means a case who was delivered at home or village health post volunteer (*Posyandu)* should be referred to health facilities or hospital for further screening or management [41]. Among the babies born at home, 14.3% cases and 43.7% controls were referred to hospital. Assessment on the level of neonatal visit showed that a percentage of inappropriate neonatal visits for cases were 4.4% compared to controls 20.8%. Inappropriate was defined as neonate who was delivered at home or village health post volunteer *(Posyandu)* but did not get the number of neonatal visit as scheduled during life period [42, 43].

Neonatal home care practice factor was evaluated using 11 items. There were 6.4% of cases had baby’s first bath immediately after delivery compared to only 2.0% of controls. Very few in both cases (1.6%) and controls (5.2%) practice neonatal bath twice a day. Higher percentages of cases (17.6%) compared to controls (0.8%) practiced Kangaroo Mother Care (KMC) during neonatal period. To warm the baby, only 3.6% of cases and 44.0% of control group used warm bottle pack. Other method of warming the baby is by using bulb lamp. This study noted that only 3.6% of cases and 15.6% of controls used bulb lamp. These practices were not popular among the cases. However, higher percentages of cases (41.2%) practice ‘*didaring*’ to warm up baby as compared to controls (10.0%). ‘*Didaring*’ is referred to activity conducted by highland population sitting near fire to warm themselves. Normally mothers after delivery will hold their babies together while sitting near the fire. Majority of both cases and controls did not apply traditional ingredients or alcohol for umbilical cord care. Both groups showed low percentage of hand washing practice before touching the babies. Late initiation of breastfeeding was higher among cases (87.6%) compared to controls (52.4%). Majority of cases (52.4%) discard the colostrums compared to controls (10.4%). There were 15.2% of cases died before starting feeding. Only 8% of cases practiced exclusive breastfeeding as compared to controls (53.2%).

Bivariate logistic regression analysis found that all 3 items under neonatal factors (sex, birth weight and gestational age) were significantly associated between neonatal mortality among LBW (p < 0.05). There were 5 items out of 10 items studied under maternal factors (maternal illness, maternal age, birth interval, antenatal care, and place of delivery) were found as significant factors for neonatal deaths. Only neonatal visit was associated with neonatal mortality under maternal and child health services. There were 8 out 11 items under neonatal care practices found associated with neonatal care (time of 1^st^ bath, bathing twice a day, KMC, the use of warm bottle pack, the use of ‘*didaring*’, initiation of breastfeeding, discarding practice of colostrums and exclusive breastfeeding).

Multivariate logistic regression was conducted to predict the final model for determinant of neonatal mortality. The model showed Cox and Snell squared test was 0.439 and Nagelkerke R Square test was 0.586. Thus it can be interpret as 44% of predictors contributing to neonatal death are explained by logistic model. There were 17 associated factors found using bivariate analysis but only 13 showed continuously significant association as predictors: sex, birth weight, gestational age, maternal illness, birth interval, antenatal care, neonatal visit, KMC, use of warm bottle pack, use of ‘*didaring*’ initiation of breastfeeding, the practice of discarding colostrums and exclusive breastfeeding. Being a boy faced 1.8 times higher risk of neonatal death than girl. Born with very low birth weight had 18 times higher risk of neonatal deaths. Preterm baby faced 1.8 times risk of mortality compared to term baby. The presence of maternal illnesses was 1.9 times risk of neonatal death (aOR 1.87(95% CI: 1.06-3.30). The birth interval showed that mothers with birth intervals less than 2 years or more that 4 years from the previous delivery faced 1.8 times high risk of having neonatal deaths (aOR 1.80, 95% CI:1.20-2.91). Inappropriate antenatal care was 2.3 times risk for neonatal mortality (aOR 2.29, 95% CI: 1.34-3.91). Having an inappropriate neonatal visit showed 7 times risk of mortality (aOR 7.04, 95% CI: 3.67-13.49). Practicing kangaroo mother care was shown as beneficial practice for low birth weight baby (aOR 15.32, 95% CI: 2.85-82.56). Those not practicing padding their babies with warm water bottle showed 21 times risk of neonatal mortality (aOR 20.7, 95% CI: 6.32-67.8). Another method found useful was using ‘*didaring*’. Those not practicing ‘*didaring*’ to warm their babies faced 4.3 times risky of neonatal mortality (aOR 4.33, 95% CI: 1.83-10.19). ‘*Didaring*’ is a method used by Acehnese people who stayed in highland to warm their body. Late initiation of breastfeeding was twice risky for neonatal mortality (aOR 2.03, 95% CI: 1.09-3.80). It is common among Acehnese to discard colostrums and this practice was 3.5 times predisposed the baby to death (aOR 3.53, 95% CI: 1.93-6.43). Not practicing exclusive breastfeeding was noted 5.6 times having the risk of neonatal mortality (aOR5.58, 95% CI: 2.89-10.77).

## Discussion

Neonatal period is crucial in determining a child’s health. Cultural health beliefs and practices are still strongly practiced especially among developing countries on mother and child care. Asian population practice postpartum traditional cultural practice to restore the balance in the body elements i.e. soil, water, wind and fire. It aims to restore general wellbeing after delivery process back to normal state [42]. Healthcare workers need to be sensitive with cultural practice among population they served. Some may be harmful and some may be beneficial to prevent mortality especially among the low birth weight baby. This study has shown that neonatal factor, maternal factor, healthcare services and neonatal care practice were determinants of neonatal mortality among low birth weight babies in Aceh. In relation to neonatal factor, the sex, birth weight group and gestational age were found significant in this study and they were similar with previous findings [10–12].

The maternal factors have a great effect on neonatal health. It is considered as direct cause of neonatal mortality [12, 30, 32, 34, 37, 43]. Maternal health refers to the health of women during pregnancy, childbirth and the postpartum period. The major direct causes of maternal illnesses include postpartum haemorrhage, maternal infection such as malaria, high blood pressure, and obstructed labour [44–47]. Therefore, the newborn’s health is closely tied to maternal health, because it is highly dependent on its mother for living. The social culture of Aceh province still put a mother as the primary caregiver at home, especially within first 40 days after birth. The father or family members are only a replacement caregiver if the mother is tired or unwell. All the baby basic needs are provided by mother for 24 hours a day, including feeding, bathing and changing diapers. If the mother is unwell or cannot care for her baby, this would affect the baby healthy or even lead to death.

After adjusting with other confounders in the multivariate logistic regression analysis, there were three variables grouped under maternal factor found as determinants of neonatal mortality among LBW: present of maternal illnesses, poor birth interval and inappropriate antenatal care. Birth interval was a determinant factor of neonatal mortality among LBW. A study done earlier in Indonesia using population data showed a strong association between short birth intervals and neonatal death [45]. Short inter pregnancy interval may result in inadequate replenishment of maternal nutrient stores and reduce foetal growth. The short inter pregnancy interval can lead to increase stress to the mother and it affects care given to her child. Shorter birth space increases the chances for a mother to develop chronic disease such as hypertension, diabetes and poor nutrition. A study done in Ethiopia showed that birth interval was strong predictor and had beneficial effects for health of mother and child [47].

Inappropriate antenatal care was found strongly associated with neonatal mortality in Aceh. According to the health system in Indonesia, all mothers in Aceh should get a minimum of 4 antenatal checks up: 1 in first trimester, 1 in second trimester and 2 in 3 trimesters. Measurement of body weight, blood pressure, symphysis fundus height tetanus toxoid vaccination and iron folic acid supplementation are minimum standard required [47]. However, 2007 IDHS documented only 84% of mothers in West Java Province attended at least 4 antenatal services [47].

In relation to maternal and child health services, it was found that inappropriate neonatal visit is seven times riskier for neonatal death. Neonatal visit is important in checking the baby’s health status and identify the risk of danger for appropriate management. According to Aceh healthcare system practice, it is recommended to have at least 2 adequate health care checks within 0–7 days and another 2 health checks around 8–28 days after delivery [47]. There are many factors that may affect the level of care in Aceh. Demographic and health survey done in Indonesia earlier has shown that poverty, poor education level, living in rural area are the reasons of having poor antenatal and postnatal health services utilization [43, 47–49].

Neonatal hypothermia is common among neonates and challenges for newborn survival. A systematic review study done earlier showed lack of thermal protection contributes to a substantial proportion of neonatal mortality globally mostly as co-morbidity of severe neonatal infection, pre term birth and asphyxia [7]. Low birth weight babies are at risk of losing body heat after delivery because of immature thermal regulation and hypoglycaemia [7]. Breastfeeding helps to warm-up the baby and giving easily digested food to replenish newborn’s need of glucose level. Early bathing has shown significantly increase of incidence of hypothermia and should be postponed until at least after 6 hour of life [50, 51]. Cultural practice of immediately bathing neonate after delivery is link with belief of ‘ritual pollution’ [52] reducing body odour in later life and helping the baby sleep and clean [53]. Using warm bottle pack helps to preserve the environment temperature. Using cultural practice ‘*didaring*’ is found beneficial in this current study among Acehnese to prevent hypothermia. It is cheap and can be used for the whole family to warm their room. This cultural practice is common among Acehnese staying in highland area. Resting near the fire may give heat to warm up but it expose to air pollution and it may affect the baby’ respiratory system.

Skin to skin care is helpful in reducing morbidity and mortality among preterm infants [54]. Practicing kangaroo mother care is shown beneficial practice to prevent neonatal deaths among Acehnese but very few of them are practicing kangaroo mother care. Awareness on its importance should be highlighted by the healthcare provider.

Improving knowledge of parent and community on neonatal care especially for low birth weight baby is very important and should be emphasised. Many cultural practices which are shared and believed to be ideal for a group of people should be tackled with caution. They need to be well informed about the danger of practicing them unless proven beneficial. Cultural practices which have been deeply rooted need to be address carefully if we are aiming on their behaviour change. Relation of cultural practice with other poor pregnancy outcome was noted in many literatures [32, 49, 55, 56].

Early initiation of breastfeeding and exclusive breastfeeding are proven evidence of protecting babies from ill and death. This study support earlier research finding on its effectiveness in neonatal survival [33]. Many studies found that cultural practice of discarding colostrums will lead to late initiation of breastfeeding [57–60]. However, once they discard the colostrums normally for 2 days only then they continue to breastfeed their baby exclusively. During the first 2 days they may start with pre lacteal feeding. Discarding colostrums is still common in Acehnese and it is significantly associated with neonatal mortality. Previous literatures have stated that this practice is performed because of believing it will cause harmful to the baby’s health. Therefore they start breastfeeding late and normally after 2 days [61–64].

In Aceh, community partnership program has been initiated since 1998 and known as ‘*Desa Siaga’.* This program helps pregnant women within their own society by arranging transports, fund and social support [47]. However, monitoring and evaluation of this program activity need to be enhanced. Harmful cultural practice should be informed for the community to recognize and prevent health problems. Ensuring appropriate health care seeking behaviour such as avoiding harmful cultural practices are important and need to be strengthened. Advocacy and dissemination of information to the community by healthcare workers will help empower their knowledge. Community based education targeting high socio cultural practices population may help to improve their awareness on obtaining appropriate antenatal and neonatal care. Each antenatal mother needs to be informed about the importance of colostrums, early initiation of breast breastfeeding and practicing exclusive breastfeeding. Lack of understanding on the important of antenatal and neonatal care may affect the neonatal survival.

Harmful cultural practice needs to be addressed as major public health priority. Any active intervention to reduce neonatal death should start at maternal health program which provides appropriate antenatal care and the child health program which provides appropriate neonatal care. Emphasising continuum of care from maternal to child health will lead to better acceptance for good health seeking behaviour. Establishing rapport with the healthcare workers will improve the community trust on changing their bad practices. The three-delays model described two decades ago can be applied in the averting neonatal death intervention strategy: early in recognition of illness, early in seeking and accessing care, and early in the provision of care once at a health facility [31]. The cost effectiveness intervention has been documented in an article published by Lancet [33]. However, cultural practice in a resilient community may affect the success of any intervention adopted. Care for mothers and neonates after birth has received little emphasis in public health programmes and, typically, has neither been monitored in demographic and health surveys nor included among key programme indicators [33]. To scale up neonatal care, two interlinked processes are required: a systematic, data-driven decision-making process and a participatory, rights-based policy process [65]. Even though Aceh has low resources, strengthening health system through proper monitoring of routine services and specific program for maternal and child health will help identifying problem to be raise up for further planning. Strategies like intervention through facility-based such as community healthcare centre (*Pukesmas*), population outreach such as village health post volunteer (*Posyandu)* and family-community partnership program *(Desa Siaga)* need to be monitored closely in term of its achievement coverage, quality, equity of services and sensitivity of cultural practice when providing services to targeted population. Involvement of men during antenatal check up, delivery and postnatal check up may open up their mind toward better decision making. Many cultural practices are implemented because of the man or their superior like mother, grandmother or strong relatives think it should be done as it is been done through their experience and this act as barrier for good health seeking behaviour [66–70].

This study has limitation in excessive accurate data on the causes of deaths, very low birth weight babies of less than 1000 g because some of them could be under reporting. Therefore, to limit incomplete information this study has focussed only moderate to low birth weight babies and causes of deaths was not included in the analysis.

KMC is not popular among Aceh and it has proven effective in prevention of neonatal deaths as found in this study and stated in the systematic review [33]. Having a group of women during antenatal care visit will allow them to learn more than they may get during individual care. They will get social support and broaden their understanding and empowered them to act positive health seeking behaviour as suggested by a study done in Canada [69]. It should be emphasized on proper healthcare provider-client communication and cultural sensitive care in order to promote usage of healthcare services as result shown in a study conducted in Ethiopia [70].

This study has its limitation as it been conducted as unmatched case control. It is well known that sex, birth weight, gestational ages and presence of maternal illnesses are determinants for neonatal mortality among the low birth weight neonates. After adjusted in the multivariate analysis, these variables persistently showed significant. Cultural beliefs and practices contribute to determinant of low birth weight in this study was inappropriate antenatal care, short birth interval, inappropriate antenatal visit, not practicing KMC, not practicing warm bottle padding, not practicing ‘*didaring*’ late initiation of breastfeeding, discard colostrums and not practicing exclusive breastfeeding. Both case and control group were comparable for family income (p > 0.05) and distance from home to capital province or district (p > 0.05). These 2 factors are important to assess to determine inappropriate of antenatal care neonatal visit received. Future explorative qualitative study should be carried out to identify gap of knowledge on people influencing neonatal care behaviour related to cultural practices.

## Conclusions

A cultural practice in this study using ‘*didaring*’ has been shown effective in preventing mortality among low birth weight in Aceh population stay in highland where source of electricity is scarce. Inappropriate neonatal visit, not practicing KMC, not practicing warm bottle pack, late initiating of breastfeeding, discarding colostrum and not practicing exclusive breastfeeding have been shown bad cultural practice that need urgent action to reduce neonatal mortality among low birth weight in Aceh population. Cultural practices affect population health seeking behaviour. Promoting good knowledge among the population and awareness on existing healthcare facilities services provided should be carried out continuously. Sensitivity to cultural practice is a key to attract population closer and practice good health behaviour. Knowledge on care of newborn especially the low birth weight baby need to be deliver to mother and family earlier during antenatal care. Healthcare workers need to conduct regular neonatal home visit to screen and identify health problem of the newborn. Existing intervention strategies should be monitored and evaluate regularly to assess its effectiveness.

## Authors’ information

RS is a medical doctor with Master of Public Health and PHD qualification. She is a maternal and neonatal public health specialist with 10 years working experience in public health sector and academician working in Universiti Kebangsaan Malaysia Medical Centre for more than 10 years. SB is a midwifery nurse with PhD qualification who has experience in the fieldwork at Aceh Province. This study was part of her thesis.

## Electronic supplementary material

Additional file 1: **STROBE Statement—Checklist of items that should be included in reports of cross-sectional studies.** (DOCX 15 KB)
