# Supplementary material for: Does cultural practice affects neonatal survival- a case control study among low birth weight babies in Aceh Province, Indonesia
Source: BMC Pregnancy Childbirth. 2014 Sep 30;14:342. doi: 10.1186/1471-2393-14-342 (PMC4262197; doi:10.1186/1471-2393-14-342)
Supplement: Supplementary file 1 — Additional file 1: STROBE Statement—Checklist of items that should be included in reports of cross-sectional studies. (DOCX 15 KB) [file 12884_2013_1250_MOESM1_ESM.docx]

STROBE Statement—Checklist of items that should be included in reports of ***cross-sectional studies***

| **recommendation** | **Comply to recommendation** |
| --- | --- |
| (*a*) Indicate the study’s design with **Title and abstract**  a commonly used term in the title or the abstract  (*b*) Provide in the abstract an informative and balanced summary of what was done and what was found | **done** |
| **Introduction**  Background/rationale  Explain the scientific background and rationale for the investigation being reported  Objectives : State specific objectives, including any prespecified hypotheses | **done** |
| **Methods**  Study design : Present key elements of study design early in the paper  Describe the setting, locations, and relevant dates, including periods of recruitment,exposure, follow-up, and data collection  Participants: Give the eligibility criteria, and the sources and methods of selection of participants  Variables : Clearly define all outcomes, exposures, predictors, potential confounders, and effect modifiers. (Give diagnostic criteria, if applicable)  Data sources/ measurement: For each variable of interest, give sources of data and details of methods of assessment (measurement).  Describe comparability of assessment methods if there is more than one group  Bias : Describe any efforts to address potential sources of bias  Study size : Explain how the study size was arrived at.  Quantitative variables: Explain how quantitative variables were handled in the analyses. (If applicable),  Describe which groupings were chosen and why  Describe all statistical methods, including those used to control for confounding  Describe any methods used to examine subgroups and interactions  Explain how missing data were addressed  If applicable, describe analytical methods taking account of sampling strategy  Statistical methods : Describe any sensitivity analyses | **Done**  **Done**  **Done**  **Done**  **Done**  **Not related**  **Done**  **Done**  **Done**  **Not related**  **Done**  **Not related**  **Done**  **Done**  **Not related** |
| **Results**  Report numbers of individuals at each stage of study—eg numbers potentially eligible, examined for eligibility, confirmed eligible, included in the study, completing follow-up, and analysed  Give reasons for non-participation at each stage  Consider use of a flow diagram  Give characteristics of study participants (eg demographic, clinical, social) and information on exposures and potential confounders  Descriptive data : Indicate number of participants with missing data for each variable of interest  Outcome data :Report numbers of outcome events or summary measures  Give unadjusted estimates and, if applicable, confounder-adjusted estimates and their precision (eg, 95% confidence interval). Make clear which confounders were adjusted for and why they were included  Report category boundaries when continuous variables were categorized  Main results :  If relevant, consider translating estimates of relative risk into absolute risk for a meaningful time period  Report other analyses done—eg analyses of subgroups and interactions, and sensitivity analyses | **Done**  **Done**  **Done**  **Done**  **Done**  **Done**  **Done**  **Done**  **Not related**  **Not done** |
| **Discussion**  Summarise key results with reference to study objectives  Discuss limitations of the study, taking into account sources of potential bias or imprecision. Discuss both direction and magnitude of any potential bias  Give a cautious overall interpretation of results considering objectives, limitations, multiplicity of analyses, results from similar studies, and other relevant evidence  Discuss the generalisability (external validity) of the study results | **done** |
| **Other information**  Give the source of funding and the role of the funders for the present study and, if  applicable, for the original study on which the present article is based  *Give information separately for exposed and unexposed | **The source of funding is from Universiti Kebangsaan Malaysia as this is part of phd thesis research project** |
